# Long-Term High Risk of Postpartum Post-Traumatic Stress Disorder (PTSD) and Associated Factors

**DOI:** 10.3390/jcm10030488

**Published:** 2021-01-30

**Authors:** Sergio Martínez-Vazquez, Julián Rodríguez-Almagro, Antonio Hernández-Martínez, Miguel Delgado-Rodríguez, Juan Miguel Martínez-Galiano

**Affiliations:** 1 Department of Nursing, Faculty of Health Sciences, University of Jaen, 23071 Jaen, Spain; sergio_sk8_9@hotmail.com (S.M.-V.); jgaliano@ujaen.es (J.M.M.-G.); 2 Department of Nursing, Physiotherapy and Occupational Therapy, Ciudad Real Faculty of Nursing, University of Castilla-La Mancha, 13071 Ciudad Real, Spain; julianj.rodriguez@uclm.es; 3 Department of Health Science, University of Jaen, 23071 Jaen, Spain; mdelgado@ujaen.es; 4 Consortium for Biomedical Research in Epidemiology and Public Health (CIBERESP), 28029 Madrid, Spain

**Keywords:** postpartum, post-traumatic stress disorder, PTSD, associated factors

## Abstract

Postpartum post-traumatic stress disorder (PTSD) is not a specific process but can last for months and may manifest itself during any subsequent pregnancies or even become chronic. This study aimed to determine the factors associated with long-term PTSD symptoms one year after delivery. A cross-sectional study was conducted on 1301 Spanish puerperal women. Data were collected on sociodemographic, obstetric, and neonatal variables. The Perinatal Post-Traumatic Stress Disorder Questionnaire (PPQ) was administered online through midwives’ associations across Spain. Crude odds ratio (OR) and adjusted odds ratio (aOR) and their 95% confidence intervals were calculated. A PPQ score ≥ 19 (high-risk) was recorded for 13.1% (171) of the participants. Identified risk factors were not respecting a birth plan (aOR = 1.89 (95% CI 1.21–2.94)), formula-feeding of the baby at discharge (aOR = 2.50 (95% CI 1.20–5.17)), postpartum surgical intervention (aOR = 2.23 (95% CI 1.02–4.85)), hospital readmission (aOR = 3.45 (95% CI 1.21–9.84)), as well as verbal obstetric violence (aOR = 3.73 (95% CI 2.52–5.53)) and psycho-affective obstetric violence (aOR = 3.98 (95% CI 2.48–6.39)). During childbirth, some clinical practices, such as formula-feeding of the newborn at discharge or types of obstetric violence towards the mother, were associated with a higher risk of PTSD symptoms one year after delivery.

## 1. Introduction

Postpartum post-traumatic stress disorder (PTSD) is a frequent disorder. Various authors have reported that approximately 50% of women who have given birth can develop it at some point after childbirth, although it can present in different forms either as a full-blown disorder or with the manifestation of one or more of its symptoms [1,2,3,4,5,6,7,8].

PTSD is not a specific process but can last for months and may manifest itself during any subsequent pregnancies or even become chronic [5,9,10]. It is a complex process with diverse symptoms; however, the most common symptom is the reenactment of the experience of the underlying cause of this disorder. Other symptoms have also been identified, such as the presence of nightmares, an opposition and denial to become a mother again, derealization, irritability, insomnia, and a lack of attachment between the mother and the baby [11,12,13,14,15,16,17,18,19,20,21].

The experience of having been exposed to a traumatic event, more specifically, to situations of abuse during childhood, has been associated with the presence of PTSD [22,23,24]. Among the obstetric variables considered as risk factors for the development of this disorder are parity, a history of spontaneous abortion, previous ectopic pregnancy, vaginal delivery, instrumental or cesarean delivery, being subjected to the Kristeller maneuver, a third- or fourth-degree perineal tear, a postpartum hemorrhage, fear of childbirth, and perceiving the treatment received during delivery assistance as incorrect by the woman giving birth [9,25,26,27,28,29,30,31].

PTSD negatively affects maternal health [15,16] and has consequences for the baby, the parents, the mother’s environment, and the family system, leading to health problems [14,16,18,32,33,34]. Regarding the newborn, maternal PTSD has been associated [35,36] with low birth weight [2]. In the immediate puerperium, it is also associated with a lower rate of initiation of breastfeeding [18]. In the long term, it has been associated with lower quality sleep for the baby and an increased risk of behavioral problems, such as a complicated or irascible temperament [37,38].

PTSD is a prevalent problem that could develop far beyond the usual postpartum care and monitoring periods. Although there is evidence that the puerperium is an important time in the onset of mental health problems, such as postpartum depression or anxiety, these problems can appear and persist from 12 or 18 months to several years after delivery [39,40]. Research on PTSD that examines patients for over a year is scarce. However, it is necessary to determine the magnitude of this problem and identify associated preventable factors to design adequate health policies that can prevent and treat pregnancy- and delivery-related PTSD. Therefore, the present study aimed to determine the factors associated with a long-term risk of PTSD in mothers a year or more after delivery.

## 2. Material and Methods

A cross-sectional study was carried out in Spain during 2019. The Research Ethics Committee of the province of Jaen approved this study with reference number TD-VCDEPP-2019/1417-N-19. Data were collected through an online survey from women who had given birth in the last 12–36 months. Before starting the questionnaire, the women were required to read an information sheet about the study and its objectives and check a box in which they confirmed their consent to participate in it; that is, they signed an ad hoc digital informed consent.

### 2.1. Design and Selection of the Subjects

The reference population was women who had given birth in Spain, in public or private hospitals or at home. To participate, the women had to have a sufficient level of instruction to read and understand Spanish. Women under 18 years of age and women with a previous psychiatric history (e.g., anxiety, depression, bipolar disorder), as well as those with a history of post-traumatic stress were excluded. To estimate the sample size required, the maximum modeling criterion was used, which requires including 10 subjects for each independent variable [41]. Taking into account that the prevalence of PTSD could be as high as 18.5% [1], a minimum of 100 women at risk of PTSD was needed to include 10 independent variables in the multivariate model and, globally, about 588 women. However, the researchers decided to include all women who met the inclusion criteria during the study period.

### 2.2. Information Sources

An online questionnaire constructed for this purpose by our research group was used and contained 64 items with open and closed questions. This questionnaire collected information on sociodemographic variables, clinical characteristics, obstetric results, clinical practices that had been performed, complaints, and opinions of the participants.

The questionnaire had been previously piloted and was distributed to the participants through the main women’s associations and the Federation of Midwives Associations of Spain (FAME), as well as its member associations that involved midwives in the dissemination of the project and the recruitment of participants. Once the study subjects were selected and agreed to participate, they were provided with the instructions to complete the questionnaire, which they then filled out in their own time. Any questions raised by the participants when completing the questionnaire could be answered via phone or a provided chat facility.

### 2.3. Study Variables

The independent variables were sociodemographic variables (maternal age, education level, current employment situation, nationality, and monthly family income in euros), obstetric variables (parity, type of delivery, induced delivery, place of delivery, admission of the newborn in a care unit, type of lactation at discharge, if it was a desired pregnancy, maternal education in preparation for childbirth classes, birth plan, if it was a single pregnancy, live newborn, type of analgesia, episiotomy, perineal tear, skin-to-skin practice, early lactation postpartum, hospital stay, postpartum surgical intervention, maternal intensive-care unit (ICU) admission, and hospital readmission), and variables related to the treatment of the participants (degree of partner support, respect received by professionals, having suffered verbal, physical or psycho-affective obstetric violence) that could potentially be related to the presence of PTSD risk.

Obstetric violence was determined through a questionnaire that evaluated different elements considered as abuse during the care received at delivery. This independent variable was decomposed to contemplate verbal, physical, and psycho-affective violence within the obstetric violence construct. This obstetric violence assessment tool has not been previously validated; however, the internal consistency of the questionnaire was analyzed using Cronbach’s alpha, obtaining a score of 0.837 for the full scale. By eliminating each item, the minimum alpha value observed was 0.818, showing good internal consistency.

The outcome variable was the presence of PTSD risk. To assess this risk of PTSD, the Perinatal Post-Traumatic Stress Disorder Questionnaire (PPQ), originally designed by Callahan, was used [42]. The PPQ consists of 14 Likert-type questions, and a score of greater than or equal to 19 points is considered as high risk. The total score of the questionnaire could vary from 0 to 56 points.

### 2.4. Statistical Analysis

First, a descriptive analysis was performed using absolute and relative frequencies for categorical variables and mean with standard deviation (SD) for quantitative variables. Next, a bivariate analysis was performed between obstetric violence and the main clinical practices and the risk of PTSD, estimating crude odds ratios (OR) and corresponding 95% confidence intervals (95% CI). Next, a multivariate analysis was carried out using binary logistic regression with the backward stepwise procedure, obtaining adjusted odds ratios (aOR) with their respective 95% Cis; *p* ˂ 0.05 was considered significant. All analyses were performed with the SPSS v24.0 statistical package (Chicago, IL USA).

## 3. Results

A total of 1301 women participated in the study. The mean age was 36.16 years (SD = 4.21 years), 57.5% (748) of the participants were over 35 years old, and 70.7% (920) of them were primiparous. Labor was induced in 40.4% (526) of the participants, 57% (741) had eutocic labor, and 25.8% (336) ended up giving birth by cesarean section (whether scheduled or emergency). The prevalence of PTSD risk (PPQ score ≥ 19) in the sample was 13.1% (171). Detailed sociodemographic data and other clinical variables can be seen in Table 1.

A bivariate analysis was performed with factors potentially associated with the risk of PTSD. A statistically significant association was observed for the following variables: maternal age, unemployment, monthly family income of between 2000 and 4000 euros, attending less than five classes in preparation for childbirth, a delivery plan, induced labor, regional anesthesia, general anesthesia, instrumental delivery, urgent cesarean section, severe tear, skin-to-skin practice, breastfeeding in the first hour, admission of the newborn to neonatal ICU (NICU), degree of partner support, feeling respected by health professionals, lactation at discharge, postpartum surgery, hospital readmission, verbal obstetric violence, physical obstetric violence, and psycho-affective obstetric violence (Table 2).

Next, a multivariate analysis was performed. Factors associated with the risk of PTSD were as follows: not respecting a birth plan (aOR = 1.89 (95% CI: 1.21–2.94)), newborn formula feeding at discharge (aOR = 2.50 (95% CI: 1.20–5.17)), postpartum surgical intervention (aOR = 2.23 [95% CI: 1.02–4.85]), maternal hospital readmission (aOR = 3.45 (95% CI: 1.21–9.84)), verbal-type obstetric violence (aOR = 3.73 (95% CI: 2.52–5.53)), and psycho-affective obstetric violence (aOR = 3.98 (95% CI: 2.48–6.39)) (Table 3).

## 4. Discussion

Approximately 13 out of 100 women were found to be at high risk for PTSD beyond the first year after delivery. Women whose birth plan was not respected, those who were formula-feeding their baby at hospital discharge, those who had to undergo postpartum surgery or be readmitted to hospital, as well as those who had experienced verbal or psycho-affective obstetric violence during labor had a higher likelihood of scoring as high-risk for PTSD on the PPQ during the late puerperium.

The population sample used in the present study was representative of the reference population. A validated instrument was used to detect the risk of PTSD [42], and this questionnaire has been used previously in populations similar to ours, both in Spain and in other countries [43]. As it is a questionnaire, there may be a selection bias associated with non-response; however, owing to the large and representative sample, we do not think that the responses of those women who did not participate would differ too much from those of the subjects in our sample. The absence of an official record noting the occurrence of obstetric violence and the self-declared nature of obstetric violence experienced by women is one of the limitations of this study, as it is a subjective experience. However, the questionnaire used for the detection of obstetric violence has a good internal consistency and has already been used in populations similar to ours. The aim of this study was to identify the presence of long-term postpartum traumatic stress. Postpartum post-traumatic stress symptoms were identified between 12 months and 36 months after childbirth; it is possible that women developed postpartum post-traumatic stress before this time, with it persisting beyond one year. It was not possible to identify the moment of onset of postpartum post-traumatic stress symptoms, but rather their presence within the assessed time period [44]. The questionnaire was presented and completed online, possibly limiting the participation of women who do not have internet access, although this is rare since smartphones, tablets, or computers are very common in the current population. The online questionnaire tool has also been included in previous research as a method of data collection [25,45].

It is important to note that the number of women who did not respond was low, not exceeding 53. The questions, and possible answers, were simple, understandable, and easy to understand for individuals of any education level, reducing the possibility of information bias to a minimum. Although the information was collected over a short period, we cannot completely rule out memory bias, but we believe that its impact on the results was minimal. Of note, the information collected appears in the women’s medical reports and in the pregnant woman’s health document (pathology during pregnancy, type of delivery, etc.) given to the participants and can be consulted any time in case of doubts. Completely ruling out a confounding bias was impossible, although attempts were made to control this in the design and in the data analysis phase, adjusting for confounding variables that could influence the outcome.

The prevalence of PTSD risk in our sample was within the median range established by various authors analyzing the PTSD risk during the puerperium (up to six weeks after delivery), ranging from 0% to 43% [1,2,3,4,5,6,7,8], although other authors, such as Vignato et al., reported a smaller range, placing it between 0.8% and 26%. While our prevalence results are within the range described in the literature, the data reported in literature correspond to PTSD prevalence during the puerperium and not in the long term, as studied in our research [46]. We were unable to compare our results with data from other authors because we did not find research that studied the prevalence of postpartum traumatic stress after 12 months of delivery. For this reason, we compared our results with data reported for the puerperium to make visible the magnitude of the problem and its persistence over time.

Having a birth plan that was not respected during clinical care was associated with the appearance of PTSD symptoms; these data coincide with those reported by Hernández-Martínez et al. [26] in their cross-sectional study carried out in Spain with almost 3000 women. This may reflect the discrepancy between the expectations that women manifest in their birth plan and the experience that they finally end up having [47]. Those unmet expectations that had been included in the birth plan may constitute an element that makes a woman live the birth experience in a different way from the one she had imagined and planned.

Newborn formula-feeding at hospital discharge was associated with the appearance of PTSD symptoms, and several authors have already studied this association [2,37]. Cook et al. found that breastfeeding was associated with lower rates of PTSD symptoms [2], in line with the results of the aforementioned authors and consistent with our findings. According to findings by Garthus-Niegel et al., the increased risk of developing PTSD occurred when breastfeeding was not started or established, making it especially important to establish both early initiation and maintenance of breastfeeding over time, even after hospital discharge [18]. The importance of maintaining this type of newborn feeding for weeks after delivery is reflected in the results of Imširagić et al. [48], showing that exclusive breastfeeding reduced the number of women at high risk of PTSD in a study conducted in Croatia and that included more than 250 women. Breastfeeding is the most recommended form of feeding for a baby. It may be that when women cannot feed their babies in this way, they feel that their expectations were not met (feeding their baby in the best possible way) and that this leads them to remember the birth process as a bad experience. In addition, mother–child emotional bond and attachment are greater with breastfeeding; this may also influence a woman’s memory of the birth as not being the best experience she had planned.

Those women who had to undergo postpartum surgery were more likely to develop PTSD. Currently, there is no research studying this association. While postpartum surgery is usually carried out to repair a structure or function to recover normality [49] and thereby improve the quality of life of the patient, the presence of injury and the sequelae derived from the intervention to repair it may increase the risk of developing PTSD.

Women who were readmitted to hospital had a higher risk of developing PTSD. There are no studies that directly relate these two variables, so we do not have data to compare with our findings. Perhaps the return to a stressful environment, such as the hospital environment, without the company of the newborn or family support [50], or the alteration of normality caused by returning to the hospital increases the risk of suffering from this disorder.

Verbal and psycho-affective obstetric violence also showed an association with the appearance of PTSD risk, with verbal violence increasing the likelihood of PTSD risk the most. Adequate verbal treatment, giving concrete and understandable information, as well as ensuring informed consent, have been found by van Dinter-Douma et al. [51] to be elements that could help reduce fear during childbirth. Obstetric violence has been little studied by authors from developed countries, making it difficult to contrast others’ results with our findings. It is striking how the treatment of professionals during delivery assistance can determine the development or maintenance of PTSD even after a significant amount of time following childbirth. Governments, such as that of Canada, have begun to collect and analyze statistics showing the importance of long-term mental disorders beyond the postpartum period, which maintain their effects for years [39]. This attention to mental health beyond the puerperium is something that various authors have pushed as a priority, highlighting pathologies that generate health problems for women nine months after delivery [52], even causing hospital readmission [53]. However, to date, we have not found research that mentions PTSD symptoms as a mental pathology in this area. For this reason, it is essential to sensitize the personnel who work in the delivery rooms of the necessary measures to ensure that a woman perceives her treatment as appropriate during her delivery. The actions of healthcare professionals who attend childbirth play a fundamental role in reducing the risk of long-term postpartum post-traumatic stress. Most of the factors identified as risk factors are related to the practice of health professionals: obstetric violence, failure to respect the birth plan, among others. For all these reasons, health professionals must be made aware of the importance of their behaviors during delivery assistance. These can be long-term risk factors for maternal pathology.

## 5. Conclusions

Women whose birth plan was not respected, those who were formula-feeding their baby at hospital discharge, those who had to undergo postpartum surgery or were readmitted to hospital, as well as those who had experienced a situation of verbal or psycho-affective obstetric violence were at higher risk of developing PTSD symptoms and maintained this risk for at least 12–36 months after delivery.

## Figures and Tables

**Table 1:** Sociodemographic and current pregnancy characteristics of the sample. C/S: cesarean section; NICU: neonatal intensive-care unit.

Variable	*n* (%)	Mean (SD)
Maternal age		36.16 (4.21)
≤35 years	553 (42.5)	
>35 years	748 (57.5)	
Education level		
Primary school	18 (1.4)	
Secondary school	51 (3.9)	
High school	273 (21.0)	
University	959 (73.7)	
Current working status		
Full-time work	558 (42.9)	
Part-time work	382 (29.4)	
Sick leave	49 (3.8)	
Unpaid leave	80 (6.1)	
Unemployed	232 (17.8)	
Nationality		
Spanish	1249 (96.0)	
Other	52 (4.0)	
Family monthly wage		
Less than 1000 euros	49 (3.8)	
Between 1000 and 2000 euros	405 (31.1)	
Between 2000 and 3000 euros	274 (21.1)	
Between 3000 and 4000 euros	108 (8.3)	
Parity		
Primiparous	920 (70.7)	
Multiparous	381 (29.3)	
Type of birth		
Normal vaginal delivery	741 (57.0)	
Instrumental	224 (17.2)	
Elective C/S	99 (7.6)	
Emergency C/S	237 (18.2)	
Induction of labor		
No	775 (59.6)	
Yes	526 (40.4)	
Place of birth		
Public hospital	1038 (79.8)	
Private hospital	230 (17.7)	
Midwife-led hospital	4 (0.3)	
Home	29 (2.2)	
Admission of the newborn to care unit		
No	1124 (86.4)	
Intermediate care	79 (6.1)	
NICU	98 (7.5)	
Infant feeding on discharge		
Maternal	1022 (78.6)	
Mixed	227 (17.4)	
Artificial	52 (4.0)	

**Table 2:** Bivariate analysis between sociodemographic or obstetric characteristics and high risk of post-traumatic stress disorder (PTSD).

Variable	High Risk of PTSD			
	Score < 19	Score ≥ 19	OR (95% CI)	*p*-Value
Maternal age				0.011
≤35 years	465 (84.1)	88 (15.9)	1 (ref.)	
>35 years	665 (88.9)	83 (11.1)	**0.66 (0.48, 0.91)**	
Education level				0.199
Primary school	13 (72.2)	5 (27.8)	1 (ref.)	
Secondary school	46 (90.2)	5 (9.8)	0.28 (0.07, 1.13)	
High school	233 (85.3)	40 (14.7)	0.45 (0.15, 1.32)	
University	838 (87.4)	121 (12.6)	0.36 (0.13, 1.07)	
Current working status				**0.019**
Full-time work	496 (88.9)	62 (11.1)	1 (ref.)	
Part-time work	337 (88.2)	45 (11.8)	1.07 (0.71, 1.61)	
Sick leave	40 (81.6)	9 (18.4)	1.80 (0.83, 3.89)	
Unpaid leave	70 (87.5)	10 (12.5)	1.14 (0.56, 2.33)	
Unemployed	187 (80.6)	45 (19.4)	**1.93 (1.27, 2.93)**	
Nationality				0.625
Spanish	1086 (86.9)	163 (13.1)	1 (ref.)	
Other	44 (84.6)	8 (15.4)	1.21 (0.56, 2.62)	
Family monthly wage				**0.001**
Less than 1000 euros	37 (75.5)	12 (24.5)	1 (ref.)	
Between 1000 and 2000 euros	335 (82.7)	70 (17.3)	0.64 (0.32, 1.30)	
Between 2000 and 3000 euros	421 (90.5)	44 (9.5)	**0.32 (0.16, 0.66)**	
Between 3000 and 4000 euros	242 (88.3)	32 (11.7)	**0.41 (0.19, 0.86)**	
More than 4000 euros	95 (88.0)	13 (12.0)	0.42 (0.17, 1.01)	
Planned pregnancy				0.328
No	76 (83.5)	15 (16.5)	1 (ref.)	
Yes	1054 (87.1)	156 (12.9)	0.75 (0.42, 1.34)	
Maternal antenatal classes				**0.005**
No	237 (90.5)	25 (9.5)	1 (ref.)	
Yes (less than 5 classes)	149 (80.1)	37 (19.9)	**2.35 (1.36, 4.07)**	
Yes (more than 5 classes)	744 (87.2)	109 (12.8)	1.39 (0.88, 2.20)	
Birth plan				**<0.001**
No	586 (88.9)	73 (11.1)	1 (ref.)	
Yes, but not respected	134 (68.7)	61 (31.3)	**3.65 (2.48, 5.39)**	
Yes, and was respected	410 (91.7)	37 (8.3)	0.72 (0.48, 1.10)	
Twin pregnancy				0.913
No	1102 (86.8)	167 (13.2)	1 (ref.)	
Yes	28 (87.5)	2 (12.5)	0.94 (0.33, 2.72)	
Live newborn				0.798
No	8 (83.3)	1 (16.7)	1 (ref.)	
Yes	1125 (86.9)	170 (13.1)	0.76 (0.09, 6.51)	
Parity				**<0.001**
Primiparous	770 (83.7)	150 (16.3)	1 (ref.)	
Multiparous	360 (94.5)	21 (5.5)	0.30 (0.19, 0.48)	
Induction of labor				**0.013**
No	688 (88.8)	87 (11.2)	1 (ref.)	
Yes	442 (84.0)	84 (16.0)	**1.50 (1.09, 2.06)**	
Natural analgesia				0.503
No	921 (86.6)	143 (13.4)	1 (ref.)	
Yes	209 (88.2)	28 (11.8)	0.86 (0.56, 1.33)	
Regional analgesia				**0.008**
No	321 (90.9)	32 (9.1)	1 (ref.)	
Yes	809 (85.3)	139 (14.7)	**1.72 (1.15, 2.59)**	
General anesthesia				**0.008**
No	1100 (87.3)	160 (12.7)	1 (ref.)	
Yes	30 (73.2)	11 (26.8)	**2.52 (1.24, 5.13)**	
Type of birth				**<0.001**
Normal vaginal delivery	678 (91.5)	63 (8.5)	1 (ref.)	
Instrumental	186 (83.0)	38 (17.0)	**2.20 (1.42, 3.39)**	
Elective C/S	88 (88.9)	11 (11.1)	1.35 (0.68, 2.65)	
Emergency C/S	178 (75.1)	59 (24.9)	**3.57 (2.41, 5.28)**	
Episiotomy				0.122
No	818 (87.8)	114 (12.2)	1 (ref.)	
Yes	312 (84.6)	57 (15.4)	1.31 (0.93, 1.85)	
Perineal tear				**<0.001**
No	715 (85.6)	120 (14.4)	1 (ref.)	
Mild	378 (91.1)	37 (8.9)	0.58 (0.40, 0.86)	
Severe	37 (72.5)	14 (27.5)	**2.26 (1.18, 4.30)**	
Skin-to-skin				**<0.001**
No	230 (72.6)	72 (23.8)	1 (ref.)	
Yes	900 (90.1)	99 (9.9)	**0.35 (0.25, 0.49)**	
Breastfeeding 1 h after childbirth				**<0.001**
No	249 (77.6)	72 (22.4)	1 (ref.)	
Yes	881 (89.9)	99 (10.1)	**0.39 (0.28, 0.54)**	
Admission of the newborn to care unit				**0.040**
No	986 (87.7)	138 (12.3)	1 (ref.)	
Intermediate care	62 (78.5)	17 (21.5)	**1.96 (1.11, 3.45)**	
NICU	82 (83.7)	16 (16.3)	1.39 (0.79, 2.45)	
Place of birth				0.124
Public hospital	903 (87.0)	135 (13.0)	1 (ref.)	
Private hospital	195 (84.8)	35 (15.2)	1.20 (0.80, 1.80)	
Midwife-led hospital	3 (75.0)	1 (25.0)	2.23 (0.23, 21.59)	
Home	29 (100.0)	0 (0.0)	0.00 (0.00, 0.00)	
Hospital length of stay				**0.039**
1 day	81 (89.0)	10 (11.0)	1 (ref.)	
2 day	580 (89.0)	72 (11.0)	1.01 (0.50, 2.03)	
3 day	269 (85.7)	45 (14.3)	1.36 (0.65, 2.81)	
4 days or more	200 (82.0)	44 (18.0)	1.78 (0.86, 3.71)	
Partner support during childbirth				**<0.001**
None	26 (78.8)	7 (21.2)	1 (ref.)	
Little	40 (62.5)	24 (37.5)	2.23 (0.84, 5.91)	
Something	97 (78.9)	26 (21.1)	0.97 (0.39, 2.55)	
Quite	271 (86.3)	43 (13.7)	0.59 (0.24, 1.44)	
A lot	696 (90.7)	71 (9.3)	**0.38 (0.16, 0.90)**	
Feeling respected by health care professionals				**<0.001**
None	19 (34.5)	36 (65.5)	1 (ref.)	
Little	99 (68.8)	45 (31.3)	**0.24 (0.12, 0.46)**	
Something	160 (76.9)	48 (23.1)	**0.16 (0.08, 0.30)**	
Quite	485 (94.4)	29 (5.6)	**0.03 (0.02, 0.06)**	
A lot	367 (96.6)	13 (3.4)	**0.02 (0.01, 0.04)**	
Infant feeding on discharge				**<0.001**
Maternal	909 (88.9)	113 (11.1)	1 (ref.)	
Mixed	185 (81.5)	42 (18.5)	**1.83 (1.24, 2.69)**	
Artificial	36 (69.2)	16 (30.8)	**3.58 (1.92, 6.65)**	
Postpartum surgical intervention				**<0.001**
No	1095 (87.6)	155 (12.4)	1 (ref.)	
Yes	35 (68.6)	16 (31.4)	**3.23 (1.75, 5.97)**	
Maternal ICU admission				NC
No	1130 (86.9)	171 (13.1)	NC	
Hospital readmission				**<0.001**
No	1114 (87.4)	160 (12.6)	1 (ref.)	
Yes	16 (59.3)	11 (40.7)	**4.79 (2.18, 10.50)**	
Verbal violence				**<0.001**
No	867 (94.3)	52 (5.7)	1 (ref.)	
Yes	263 (68.8)	119 (31.2)	**7.54 (5.30, 10.75)**	
Physical violence				**<0.001**
No	448 (93.3)	32 (6.7)	1 (ref.)	
Yes	682 (83.1)	139 (16.9)	**2.85 (1.91, 4.27)**	
Psych-affective violence				**<0.001**
No	710 (96.2)	29 (3.8)	1 (ref.)	
Yes	420 (74.6)	143 (25.4)	**8.63 (5.66, 13.17)**	
Violence (dichotomous)				**<0.001**
No	324 (98.2)	6 (1.8)	1 (ref.)	
Yes	806 (83.0)	165 (17.0)	11.06 (4.85, 25.22)	

OR: odds ratio; NC: not calculated; ICU: intensive-care unit. Statistically significant values in bold.

**Table 3:** Multivariate analysis between sociodemographic or obstetric characteristics and high risk of PTSD.

Variable	High Risk of PTSD and Associated Factors			
	Score < 19	Score ≥ 19	aOR * (95% CI)	*p*-Value
Birth plan				
No	586 (88.9)	73 (11.1)	1 (ref.)	
Yes, but not respected	134 (68.7)	61 (31.3)	**1.89 (1.21, 2.94)**	**0.005**
Yes, and was respected	410 (91.7)	37 (8.3)	1.10 (0.69, 1.73)	0.710
Infant feeding on discharge				
Maternal	909 (88.9)	113 (11.1)	1 (ref.)	
Mixed	185 (81.5)	42 (18.5)	1.24 (0.80, 1.93)	0.344
Artificial	36 (69.2)	16 (30.8)	**2.50 (1.20, 5.17)**	**0.014**
Postpartum surgical intervention				
No	1095 (87.6)	155 (12.4)	1 (ref.)	
Yes	35 (68.6)	16 (31.4)	**2.23 (1.02, 4.85)**	**0.024**
Hospital readmission				
No	1114 (87.4)	160 (12.6)	1 (ref.)	
Yes	16 (59.3)	11 (40.7)	**3.45 (1.21, 9.84)**	**0.020**
Verbal violence				
No	867 (94.3)	52 (5.7)	1 (ref.)	
Yes	263 (68.8)	119 (31.2)	**3.73 (2.52, 5.53)**	**<0.001**
Psych-affective violence				
No	710 (96.2)	29 (3.8)	1 (ref.)	
Yes	420 (74.6)	143 (25.4)	**3.98 (2.48, 6.39)**	**<0.001**

* Odds Ratio adjusted after multivariate analysis using the backward stepwise procedure of Binary Logistic Regression. Statistically significant values in bold.

## Data Availability

The datasets used and/or analysed during the current study are available from the corresponding author on reasonable request.
